# General and event‐related psychological stress, and suicidal ideation among hospital workers during the coronavirus disease 2019 pandemic: Findings from the third wave of repeated cross‐sectional studies

**DOI:** 10.1002/pcn5.70157

**Published:** 2025-07-14

**Authors:** Keiko Ide, Akira Suda, Asuka Yoshimi, Junichi Fujita, Munetaka Nomoto, Masatoshi Miyauchi, Tomohide Roppongi, Akitoyo Hishimoto, Toshinari Odawara, Takeshi Asami

**Affiliations:** ^1^ Department of Psychiatry Yokohama City University School of Medicine Yokohama Japan; ^2^ Psychiatric Center Yokohama City University Medical Center Yokohama Japan; ^3^ Department of Child Psychiatry Yokohama City University Hospital Yokohama Japan; ^4^ Department of Psychiatry Kobe University Graduate School of Medicine Kobe Japan; ^5^ Health Management Center Yokohama City University Yokohama Japan

**Keywords:** COVID‐19, health personnel, psychological stress, stress disorders, suicidal ideation

## Abstract

**Aim:**

The psychological impact of the coronavirus disease 2019 (COVID‐19) pandemic on hospital workers has been reported, but most previous studies focused on the first year of the pandemic, and long‐term monitoring remains scarce. This study aimed to evaluate the psychological status of hospital workers as of March 2023, and identify associated factors.

**Methods:**

A cross‐sectional survey was conducted in March 2023 among all workers at two university hospitals in Yokohama, Japan. Similar surveys were conducted in March–April 2020 and March 2021. The prevalence of general psychological stress, event‐related stress, and suicidal ideation was assessed using the 12‐item General Health Questionnaire (GHQ‐12), the Impact of Event Scale‐Revised (IES‐R), and Item 9 of the Patient Health Questionnaire (PHQ‐9), respectively. Multivariable logistic regression analysis was performed to identify associated factors.

**Results:**

A total of 4244 questionnaires were distributed and 2635 responses (62.1%) were analyzed. Severe general stress, event‐related stress, and suicidal ideation were observed in 38.0%, 18.1%, and 10.0% of participants, respectively. Regression analysis identified isolation, exhaustion, and being office workers or support staff as significant factors for general stress; living with a partner and feeling protected were inversely associated. Event‐related stress was associated with clerical work, isolation, and exhaustion. Suicidal ideation was associated with younger age, isolation, and coexisting general and event‐related stress, while the anxiety factor showed a negative association.

**Conclusion:**

This study highlights the sustained psychological burden experienced by hospital workers in 2023. The findings underscore the importance of strategies to reduce isolation and enhance mental health support in healthcare settings.

## INTRODUCTION

The psychological impact of the coronavirus disease 2019 (COVID‐19) outbreak on hospital workers has been widely reported, particularly during the first year of the pandemic.^1^, ^2^, ^3^, ^4^, ^5^ Most studies indicate that the mental state of healthcare workers was significantly affected during the COVID‐19 pandemic. Previous pandemics, such as the 2003 severe acute respiratory syndrome (SARS) outbreak, have also been shown to cause long‐lasting psychological effects among healthcare workers.^6^, ^7^, ^8^ However, the global scale and prolonged duration of the COVID‐19 pandemic present unprecedented challenges, underscoring the importance of understanding its long‐term mental health consequences.

After multiple waves across various countries, the pandemic has now subsided; however, as an unprecedented, prolonged global infectious disease outbreak, it remains crucial to assess the extent of its psychological impact on healthcare workers and how this impact has evolved over time to better prepare for future pandemics. Despite this importance, few studies have continuously tracked changes in the psychological well‐being of healthcare workers throughout the course of the COVID‐19 pandemic. Notably, the majority of studies have employed a cross‐sectional design, assessing mental health at only one or two time points, whereas longitudinal studies or those conducting repeated surveys three or more times are relatively scarce.^9^, ^10^, ^11^

Furthermore, reports have indicated an increase in suicide rates in Japan following the onset of the COVID‐19 pandemic,^12^, ^13^ despite a decline in suicide rates over the preceding decade. Suicidal ideation is a known risk factor for suicide,^14^, ^15^ and it has been reported that the deterioration of mental health during the pandemic has contributed to an increase in suicidal ideation.^16^ Therefore, it is essential to investigate whether the worsening mental state of healthcare workers during the pandemic has led to an increased prevalence of suicidal ideation.

We conducted repeated cross‐sectional surveys to assess the psychological impact of the pandemic on workers at two university hospitals in Yokohama,^17^, ^18^ both of which began admitting COVID‐19‐infected patients during the major outbreak aboard the *Diamond Princess* cruise ship (anchored at the Port of Yokohama) in the early stages of the pandemic in Japan. The first survey, conducted at the beginning of the outbreak, revealed that hospital workers experienced severe psychological stress in addition to physical exhaustion. The second survey, conducted 1 year later, indicated a further deterioration in their psychological well‐being. These findings led us to hypothesize that psychological stress and suicidal ideation among hospital workers might have persisted or even worsened throughout the pandemic.

To systematically evaluate these changes, we administered nearly identical survey questions to all staff members working in hospitals admitting COVID‐19 patients, allowing us to assess the progression of mental health impacts over time. This study was conducted at three key time points: during the initial phase of the pandemic, 1 year later, and 3 years later. In this study, we present findings on changes in the mental health status of hospital workers over a 3‐year period, incorporating the most recent research results. The objective of this study is to evaluate the long‐term psychological trends among hospital workers exposed to the COVID‐19 pandemic and to explore appropriate measures for supporting hospital staff in managing event‐related psychological stress.

## METHODS

### Study design and setting

This study employed a repeated cross‐sectional observational design to assess the psychological impact of the COVID‐19 pandemic on hospital workers at Yokohama City University Hospital and Yokohama City University Medical Center. The surveys were administered in three waves: March–April 2020,^17^ March 2021,^18^ and March 7–24, 2023. As in the previous rounds of this study, the same survey methodology was consistently applied, reinforcing the value of repeated assessments over time.

Paper‐based, self‐administered, anonymous questionnaires were distributed to all hospital staff. These were either handed directly to individuals, placed on their desks, or delivered via the hospital's internal mail system. To maximize response rates and inclusivity, all staff members, including those without Internet access or familiarity with online platforms, had the opportunity to participate. Maintaining a uniform approach across all three survey periods ensured comparability of data and enhanced the reliability of findings.

### Participants

All individuals employed at Yokohama City University Hospital and Yokohama City University Medical Center during each survey period were invited to participate. This included physicians, nurses, and a comprehensive range of allied health professionals, administrative personnel, and support staff. A detailed breakdown of job categories is provided in Table 1. The inclusion of all staff roles aimed to comprehensively assess the psychological impact of the COVID‐19 pandemic across the diverse occupational structure of the hospital environment. Participation was strictly voluntary.

**Table 1:** Participants' characteristics associated with severe general stress, severe event‐related stress, and suicidal ideation in the third survey result.

	Overall		Severe general stress			Severe event‐related stress			Suicidal ideation		
	*N*	%	*n*	%	*p* value	*n*	%	*p* value	*n*	%	*p* value
Total	2635		1001	38.0%		478	18.1%		263	10.0%	
Gender											
Men	606	23.0%	168	16.8%		82	17.2%		56	21.3%	
Women	2029	77.0%	833	83.2%	<0.001**	396	82.8%	<0.001**	207	78.7%	0.537
Age, years											
≥29	661	25.1%	254	25.4%		108	22.6%		76	28.9%	
30–39	645	24.5%	221	22.1%		99	20.7%		70	26.6%	
40–49	646	24.5%	255	25.5%		122	25.5%		64	24.3	
≤50	683	25.9%	271	27.1%	0.152	149	31.2%	0.010*	53	20.2%	0.112
Occupation											
Medical doctor	474	18.0%	116^a^	24.5%		49^a^	10.3%		35^a^	7.4%	
Nurse	1106	42.0%	453^a^	41.0%		198^b^	17.9%		101^b^	9.1%	
Other medical professional	375	14.2%	135^b^	36.0%		65^b^	17.3%		44^b^	11.7%	
Office worker/Clinical clerk	561	21.3%	245^a^	43.7%		137^a^	24.4%		71^a^	12.7%	
Other support staff	119	4.5%	52^b^	43.7%	<0.001**	29^b^	24.4%	<0.001**	12^b^	10.1%	0.037*
Preexisting disease											
Yes	236	9.0%	109	10.9%		58	12.1%		27	10.3%	
No	2399	91.0%	892	89.1%	0.007**	420	87.9%	0.007**	236	89.7%	0.433
Living with partners											
Yes	1445	54.8%	491	49.1%		230	48.1%		120	45.6%	
No	1190	45.2%	510	50.9%	<0.001**	248	51.9%	0.001**	143	54.4%	0.002**
Living with elderly											
Yes	314	11.9%	147	14.7%		85	17.8%		40	15.2%	
No	2321	88.1%	854	85.3%	<0.001**	393	82.2%	<0.001**	223	84.8%	0.082
Confident in standard precaution											
Yes	1761	66.8%	625	62.4%		309	64.6%		163	62.0%	
No	874	33.2%	376	37.6%	<0.001**	169	35.3%	0.262	100	38.0%	0.078
Direct contact with COVID‐19 patients											
Yes	2075	78.7%	777	77.6%		357	74.7%		194	73.8%	
No or don't know	560	21.3%	224	22.4%	0.281	121	25.3%	0.019*	69	21.3%	0.039*

*Note*: Superscript letters (a, b) indicate subsets of categories whose column proportions do not differ significantly from each other at the 0.05 level (Bonferroni‐adjusted comparison).

Abbreviation: COVID‐19, coronavirus disease 2019.

* *p* < 0.05;

** *p* < 0.01.

### Data source and questionnaire content

Before completing the questionnaire, participants provided informed consent by checking a designated box. From the second survey onward, an additional question was introduced to ascertain whether respondents had participated in previous waves of the study. This allowed for an estimation of the proportion of participants who had completed multiple surveys, acknowledging that some individuals may have transferred, started new positions, or resigned. Due to the anonymity of responses, individual data could not be linked across time points.

The initial survey comprised four sections: sociodemographic characteristics, the 12‐item General Health Questionnaire (GHQ‐12),^19^, ^20^ the Impact of Event Scale‐Revised (IES‐R),^21^, ^22^ and COVID‐19‐related stress measures. Starting from the second period, the ninth item of the Patient Health Questionnaire (PHQ‐9)^23^, ^24^ was added to assess suicidal ideation.

Sociodemographic information included age, gender, occupation, preexisting health conditions, living arrangements (e.g., whether the respondent lived with a partner or an elderly individual), confidence in standard infection‐control precautions, and direct exposure to COVID‐19 patients. These variables were considered potential risk factors for COVID‐19‐related psychological stress.

To assess general psychological stress, the GHQ‐12 total score was calculated using the GHQ scoring method.^19^ Workers were classified into two groups based on a threshold score of 3/4.^25^ The IES‐R was used to evaluate event‐related psychological stress linked to the COVID‐19 pandemic. A score of 25 or higher was considered indicative of significant stress.^26^, ^27^, ^28^ Suicidal ideation was assessed using the PHQ‐9 item assessing suicidal thoughts: “Over the last two weeks, how often have you been bothered by thoughts that you would be better off dead or hurting yourself in some way?” This item was rated on a four‐point Likert scale, and responses of 1 or greater were classified as indicative of suicidal ideation, following established guidelines.^29^, ^30^, ^31^ Prior studies have established that responses to the ninth item of the PHQ‐9 serve as a reliable indicator of increased risk for suicide attempts or death.^32^

To assess pandemic‐related stressors, a modified GHQ scoring method^19^ was applied to evaluate the 26 questions, assigning scores as follows: *never/rarely* = 0, *sometimes/always* = 1. Based on their exposure to specific stressors, workers were categorized accordingly. Of these, 19 items were derived from previous research on past outbreaks,^33^, ^34^ while seven newly developed questions were incorporated to address additional relevant aspects. These 26 items were identical across all three surveys, preserving methodological consistency and allowing for a robust comparison of stress factors over time. The internal reliability of the 26‐item scale, as measured by Cronbach's *α* coefficient (*α* = 0.83), demonstrated strong internal consistency.

### Statistical methods

Descriptive statistics were employed to summarize participant characteristics and estimate the prevalence of general stress, event‐related stress, and suicidal ideation. *χ* ^2^ tests were used to examine associations between sociodemographic factors and each type of stress within the 2023 data set. Comparisons across the three survey waves were presented descriptively, without statistical testing, due to differences in the study populations.

To further explore the structure of pandemic‐related stressors, factor analysis was performed on the 26 stress‐related items. Multivariable logistic regression analysis was conducted to identify variables significantly associated with psychological stress and suicidal ideation. All statistical analyses were performed using IBM SPSS Statistics (Version 21.0; IBM Corp.) with a significance threshold of *p* < 0.05 (two‐tailed).

By employing the same methodological framework across multiple time points, this study ensures a reliable assessment of long‐term psychological trends among hospital workers exposed to the COVID‐19 pandemic. The consistency in survey content and administration strengthens the validity of the findings, emphasizing the importance of sustained monitoring in occupational mental health research.

## RESULTS

In the third survey, questionnaires were distributed to 4244 hospital workers, and 2800 questionnaires were collected from participants who had provided informed consent (66%). Of these questionnaires, 165 were excluded because of missing data for at least one sociodemographic characteristic or psychological rating scale. Therefore, 2635 questionnaires (62.1%) were used in the analyses; 33.9% and 34.6% of the respondents had also responded to the first and second questionnaires, respectively (Table 1).

**Figure 1:**
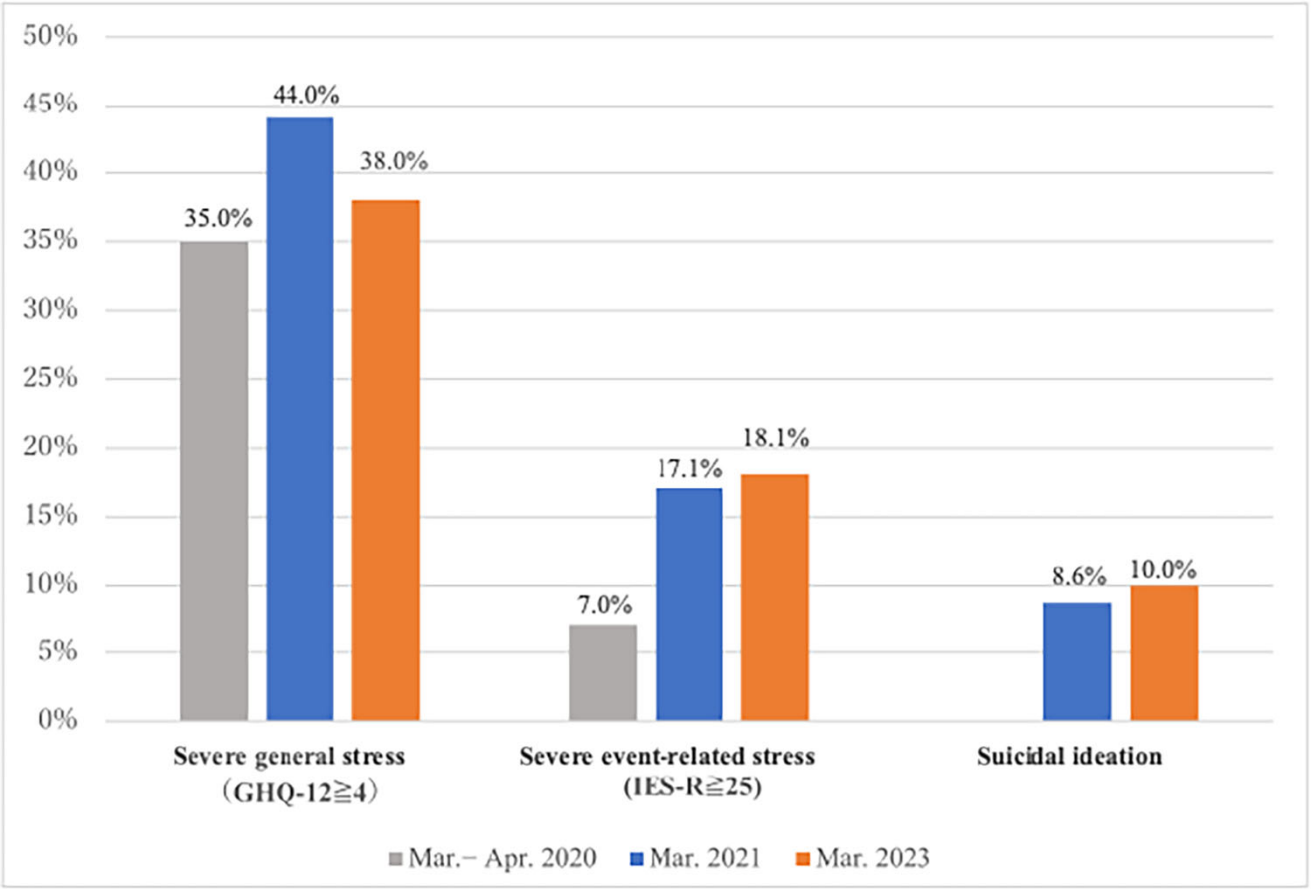
Morbidity rates of severe general stress, severe event‐related stress, and suicidal ideation. The figure shows the percentage of hospital workers reporting severe general psychological stress (12‐item General Health Questionnaire [GHQ‐12] score ≥ 4), severe event‐related stress (Impact of Event Scale‐Revised [IES‐R] score ≥ 25), and suicidal ideation (based on Patient Health Questionnaire [PHQ‐9] Item 9) in the first (2020), second (2021), and third (2023) surveys. Suicidal ideation was not assessed in the 2020 survey. The values represent descriptive comparisons across independent samples.

The prevalence rate of severe general stress (GHQ‐12 score ≥4) was 38.0%, compared with 35.0% in the first period and 44.0% in the second period. That of severe event‐related stress (IES‐R score ≥25) was 18.1%, compared with 7.0% and 17.1%, respectively. Suicidal ideation was reported by 10.0% of participants, compared with 8.6% in the second period (not measured in the first period). These values are based on descriptive comparisons across independent samples (Figure 1). To aid in interpretation, Table 2 summarizes key demographic and psychological characteristics across the three survey waves, including prevalence rates of stress indicators and the composition of participant groups.

**Table 2:** Comparison of psychological and demographic characteristics across the three survey waves (2020, 2021, and 2023).

Category	2020 (1st survey)	2021 (2nd survey)	2023 (3rd survey)
Total participants	2697	2813	2635
Severe general stress (%)	944 (35.0%)	1237 (44.0%)	1001 (38.0%)
Severe event‐related stress (%)	189 (7.0%)	480 (17.1%)	478 (18.1%)
Suicidal ideation (%)	—	243 (8.6%)	263 (10.0%)
Gender			
Women (%)	1995 (74.0%)	2078 (73.9%)	2029 (77.0%)
Age			
≤29 years (%)	625 (23.2%)	667 (23.7%)	661 (25.1%)
30–39 years (%)	707 (26.2%)	727 (25.8%)	645 (24.5%)
40–49 years (%)	750 (27.8%)	737 (26.2%)	646 (24.5%)
≥50 years (%)	615 (22.8%)	682 (24.2%)	683 (25.9%)
Occupation			
Medical doctor (%)	555 (20.6%)	588 (20.9%)	474 (18.0%)
Nurse (%)	1045 (38.7%)	1055 (37.5%)	1106 (42.0%)
Other medical professional (%)	359 (13.3%)	390 (13.9%)	375 (14.2%)
Office worker/Clinical clerk (%)	527 (19.5%)	548 (19.5%)	561 (21.3%)
Other support staff (%)	211 (7.8%)	232 (8.2%)	119 (4.5%)
Preexisting disease (%)	191 (7.1%)	264 (9.4%)	236 (9.0%)
Living with partner (%)	1422 (52.7%)	1539 (54.7%)	1445 (54.8%)
Living with elderly (%)	363 (13.5%)	362 (12.9%)	314 (11.9%)
Direct COVID‐19 contact (%)	328 (12.2%)	1172 (41.7%)	2075 (78.7%)

*Note*: Data from the 2020 and 2021 surveys are derived from previously published reports.^17^, ^18^

Abbreviation: COVID‐19, coronavirus disease 2019.

Factor analysis of the questions on COVID‐19‐related stress extracted six factors: “isolation,” “anxiety,” “workload,” “exhaustion,” “uncertainty,” and the “feeling of being protected” (Table 3).

**Table 3:** Factor analysis of the 26 stress‐related questions in the third survey result.

Questions		F1	F2	F3	F4	F5	F6
*Factor 1: Isolated (Cronbach's α = 0.65)*							
Q14	**Feeling of being isolated**	**0.525**	−0.073	−0.006	−0.021	0.076	−0.041
Q25	**Unfairness**	**0.515**	−0.023	0.122	0.032	−0.014	−0.14
Q13	**Hesitation to work**	**0.479**	0.02	0.1	0.039	−0.021	−0.126
Q23	**Increase of Internet and SNS exposure**	**0.466**	0.143	−0.105	−0.005	−0.066	0.121
Q22	**Increase of TV exposure**	**0.442**	0.116	−0.11	−0.042	−0.06	0.159
Q16	**Insomnia**	**0.424**	−0.082	−0.012	0.155	0.007	0.025
Q24	Greater amount of alcohol drinking	0.394	−0.006	0.015	−0.038	−0.13	0.037
Q26	Doubt about uninformed serious information	0.363	0.024	−0.037	−0.025	0.212	−0.068
Q8	Feeling of being avoided by others	0.311	−0.05	0.039	−0.105	0.185	−0.016
Q15	Elevated mood	0.274	−0.07	−0.007	0.006	0.012	0.175
*Factor 2: Anxiety (Cronbach's α = 0.80)*							
Q2	**Anxiety about infecting family**	−0.033	**0.856**	0.015	−0.011	−0.059	−0.057
Q1	**Anxiety about being infected**	−0.041	**0.817**	0.036	−0.041	−0.004	−0.05
Q21	Heightened awareness of physical condition management	0.067	0.397	0.028	0.034	0.016	0.197
Q5	Anxiety of being infected during commuting	0.01	0.353	−0.06	−0.002	0.264	0.026
Q12	Anxiety about compensation	0.192	0.218	−0.027	0.084	0.139	−0.086
*Factor 3: Workload (Cronbach's α = 0.81)*							
Q3	**Burden of increased quantity of work**	−0.044	0.009	**0.871**	0.013	−0.034	0.04
Q4	**Burden of change of quality of work**	−0.007	0.029	**0.794**	−0.02	0.045	0.021
Q20	Feeling of having no choice but to work due to obligation	0.109	0.16	0.178	0.082	−0.007	0.002
*Factor 4: Exhaustion (Cronbach's α = 0.85)*							
Q17	**Physical exhaustion**	−0.04	−0.02	0.018	**0.895**	−0.004	0.038
Q18	**Mental exhaustion**	0.043	0.012	−0.014	**0.833**	0.004	−0.02
*Factor 5: Unknowns (Cronbach's α = 0.71)*							
Q6	**Unknowns of means to prevent from infection**	−0.057	−0.069	0.009	0.019	**0.806**	0.024
Q7	**Unknowns of infectivity and virulence**	−0.052	0.142	0.006	−0.007	**0.711**	0.026
*Factor 6: Feeling of being protected* (*Cronbach's α* = *0.57*)							
Q10	**Feeling of being protected by hospital**	0.04	−0.005	0.023	0.004	0.012	**0.687**
Q9	**Feeling of being protected by national and local governments**	0.117	−0.08	0.058	−0.056	0.028	**0.585**
Q19	Motivation to work	−0.077	0.064	−0.018	0.07	0.002	0.377
Q11	Feeling of being supported by family	0.046	0.186	0.034	0.042	0.013	0.267
	* *Eigenvalue	5.667	1.956	1.597	1.497	1.268	1.017
	* *Variance explained (%)	21.796	7.522	6.143	5.759	4.876	3.911
	* *Between‐factor correlation						
	* *F2	0.48					
	* *F3	0.48	0.465				
	* *F4	0.606	0.461	0.556			
	* *F5	0.55	0.567	0.344	0.353		
	* *F6	−0.206	−0.055	−0.218	−0.194	−0.129	

*Note*: Bold values indicate factor loading ≥0.40. Cronbach's *α* was computed without excluded items.

Abbreviation: F, Factor.

Multivariable logistic regression analysis identified several factors associated with severe general stress, event‐related stress, and suicidal ideation (Table 4).

**Table 4:** Factors associated with severe general stress, severe event‐related stress, and suicidal ideation in the third survey result.

	Severe general stress		Severe event‐related stress		Suicidal ideation	
	aOR (95% CI)	*p* value	aOR (95% CI)	*p* value	aOR (95% CI)	*p* value
Gender, female versus male	1.03 (0.79–1.34)	0.81	0.77 (0.54–1.09)	0.14	0.82 (0.54–1.24)	0.35
Age group (years), versus ≥50 years		0.33		0.18		0.004**
≤29	0.96 (0.72–1.28)	0.79	0.71 (0.49–1.02)	0.07	2.28 (1.42–3.67)	<0.001**
30–39	0.95 (0.72–1.25)	0.70	0.74 (0.52–1.05)	0.09	2.05 (1.30–3.21)	0.002**
40–49	1.18 (0.91–1.53)	0.22	0.93 (0.67–1.29)	0.68	1.53 (0.99–2.37)	0.06
Occupation, versus medical doctor		0.002**		0.008**		0.291
Nurse	1.05 (0.77–1.44)	0.76	0.94 (0.60–1.46)	0.78	0.77 (0.46–1.30)	0.32
Other medical professional	1.34 (0.95–1.91)	0.10	1.33 (0.83–2.16)	0.24	1.18 (0.68–2.05)	0.56
Office worker/clinical clerk	1.71 (1.20–2.43)	0.003**	1.67 (1.04–2.70)	0.04*	1.13 (0.65–1.98)	0.66
Other support staff	1.86 (1.11–3.12)	0.02*	1.74 (0.90–3.36)	0.10	1.17 (0.51–2.69)	0.71
Preexisting disease	1.46 (1.07–2.01)	0.02*	1.19 (0.81–1.74)	0.38	1.09 (0.67–1.78)	0.74
Living with partner	0.80 (0.66–0.98)	0.03*	0.86 (0.67–1.11)	0.24	0.98 (0.71–1.34)	0.89
Confident in standard precaution	1.27 (1.05–1.54)	0.01*	0.87 (0.68–1.11)	0.26	0.97 (0.71–1.30)	0.81
Factor 1: Isolation	1.36 (1.27–1.46)	<0.001**	1.51 (1.39–1.64)	<0.001**	1.22 (1.10–1.36)	<0.001**
Factor 2: Anxiety	0.98 (0.87–1.11)	0.76	1.03 (0.87–1.21)	0.76	0.73 (0.60–0.89)	0.002**
Factor 3: Workload	1.00 (0.89–1.13)	0.998	0.92 (0.78–1.08)	0.30	0.93 (0.77–1.13)	0.47
Factor 4: Exhaustion	2.20 (1.94–2.50)	<0.001**	1.83 (1.50–2.23)	<0.001**	1.24 (0.98–1.58)	0.08
Factor 5: Uncertainty	0.97 (0.85–1.10)	0.63	1.12 (0.96–1.31)	0.14	1.00 (0.82–1.22)	0.996
Factor 6: Protected	0.86 (0.74–0.99)	0.03*	0.99 (0.82–1.20)	0.93	1.08 (0.85–1.36)	0.54
Severe general stress			3.22 (2.51–4.13)	<0.001**	7.13 (4.86–10.5)	<0.001**
Severe event‐related stress					2.57 (1.88–3.53)	<0.001**

Abbreviations: aOR, adjusted odds ratio; CI, confidence interval.

* *p* < 0.05;

** *p* < 0.01.

For severe general stress, significant associations were observed with being office workers/clinical clerks (adjusted odds ratio [aOR] = 1.71, 95% confidence interval [CI]: 1.20–2.43, *p* = 0.003), being other support staff (aOR = 1.86, 95% CI: 1.11–3.12, *p* = 0.02), having preexisting diseases (aOR = 1.46, 95% CI: 1.07–2.01, *p* = 0.02), and the isolation (aOR = 1.36, 95% CI: 1.27–1.46, *p* < 0.001) and exhaustion factors (aOR = 2.20, 95% CI: 1.94–2.50, *p* < 0.001). In contrast, living with a partner (aOR = 0.80, 95% CI: 0.66–0.98, *p* = 0.03) and the feeling of being protected (aOR = 0.86, 95% CI: 0.74–0.99, *p* = 0.03) were inversely associated with general stress.

For severe event‐related stress, significant associations were found with being office workers/clinical clerks (aOR = 1.67, 95% CI: 1.04–2.70, *p* = 0.04), as well as higher scores on the isolation (aOR = 1.51, 95% CI: 1.39–1.64, *p* < 0.001) and exhaustion factors (aOR = 1.83, 95% CI: 1.50–2.23, *p* < 0.001).

For suicidal ideation, significant associations were observed with younger age groups: individuals aged ≤29 years (aOR = 2.28, 95% CI: 1.42–3.67, *p* < 0.001) and those aged 30–39 years (aOR = 2.05, 95% CI: 1.30–3.21, *p* = 0.002), compared with those aged ≥50 years. The isolation factor was also associated with increased suicidal ideation (aOR = 1.22, 95% CI: 1.10–1.36, *p* < 0.001), while the anxiety factor showed a negative association (aOR = 0.73, 95% CI: 0.60–0.89, *p* = 0.002). Additionally, comorbid general or event‐related stress showed strong associations with suicidal ideation: general stress (aOR = 3.22, 95% CI: 2.51–4.13, *p* < 0.001) and event‐related stress (aOR = 7.13, 95% CI: 4.86–10.5, *p* < 0.001).

## DISCUSSION

This study assessed the psychological status of hospital workers in 2023, approximately 3 years after the onset of the COVID‐19 pandemic. In the cross‐sectional survey, 38.0% of participants reported severe general distress, 18.1% reported severe event‐related distress, and 10.0% reported suicidal ideation. These results indicate that, even in 2023, a substantial proportion of healthcare workers continued to experience psychological symptoms, such as stress and suicidal thoughts. Given the use of independent samples, these results should be interpreted as a descriptive snapshot of the mental health status of hospital workers during the later phase of the pandemic.

Although several studies have examined the early psychological effects of the COVID‐19 pandemic, few have assessed its long‐term trajectory among healthcare workers in Japan and globally. Our findings are consistent with previous reports suggesting that healthcare workers are at risk for prolonged psychological distress.^10^, ^35^ For example, research on the 2003 SARS outbreak demonstrated that its psychological impact persisted for at least 3 years.^6^, ^7^, ^8^ While the COVID‐19 pandemic has lasted longer and has had a broader societal impact than SARS, it remains necessary to continue monitoring mental health among healthcare workers—regardless of whether the causes are pandemic‐specific or reflect ongoing structural challenges within healthcare systems.

Among the demographic factors examined in this study, being in one's 20s or 30s was identified as an associated factor for suicidal ideation. This finding is consistent with previous research, which has consistently demonstrated that suicidal ideation is more prevalent among younger individuals.^36^ Moreover, multiple studies across different countries have reported that the COVID‐19 pandemic has exacerbated this issue, with suicide‐related distress among young people remaining elevated even after the initial phase of the pandemic.^37^, ^38^ Several reports indicate that suicidal ideation among young individuals increased after the onset of the pandemic and has yet to return to pre‐pandemic levels. While research on post‐pandemic mental health among younger populations remains limited, emerging evidence suggests that their psychological well‐being has not fully recovered.^39^, ^40^, ^41^ In fact, recent findings point to a further deterioration in adolescent and young adult mental health, raising concerns about the potential long‐term psychological consequences of the pandemic.

Additionally, previous studies have highlighted disproportionately high suicide rates among nurses in the United States,^42^ and research has indicated that the psychological impact of COVID‐19 has been more pronounced among nonmedical professionals, young individuals, and women.^10^, ^43^, ^44^ However, in the present study, we did not observe statistically significant differences in suicidal ideation based on job type or gender. Nevertheless, levels of general psychological stress and traumatic event‐related stress—both known associated factors for suicidal ideation—varied across occupational groups. Specifically, nonmedical hospital staff, including administrative personnel and support workers, exhibited higher levels of both general psychological stress and traumatic event‐related stress compared to medical doctors. These findings suggest that the psychological burden of the COVID‐19 pandemic has persisted not only among frontline healthcare workers but also among hospital staff in nonclinical roles. Given that nonmedical personnel may have less access to mental health resources and peer support within healthcare settings, future research should explore targeted interventions to address their specific needs.

In this study, exhaustion and workload emerged as significant associated factors for general psychological stress, which, in turn, was identified as an associated factor for traumatic event‐related stress and suicidal ideation. Social isolation also played a critical role, as it was associated with all three outcomes. Previous research^45^ has demonstrated that traumatic stress and loneliness exacerbated mental health issues among healthcare workers during the COVID‐19 pandemic. Furthermore, studies conducted among the general population in Japan reported similar findings, indicating that persistent loneliness and prolonged social isolation remained unresolved throughout the pandemic.^46^ These challenges became increasingly shaped by a broader and more intricate set of influences over time, further compounding their impact on mental health.

The finding that the “anxiety” factor was negatively associated with suicidal ideation may initially appear paradoxical. However, previous studies conducted during the COVID‐19 pandemic have indicated that concern about infection does not necessarily exacerbate psychological distress and may, in some cases, facilitate adaptive coping behaviors. For instance, Sasaki et al.^47^ found that employees in organizations implementing more extensive infection‐control measures reported lower psychological stress despite higher levels of concern about COVID‐19. Similarly, Kawakami et al.^48^ reported that downloading the government‐issued contact‐tracing app (COCOA) was associated with reduced psychological distress, even though infection‐related worry remained unchanged. These findings suggest that recognizing and actively responding to anxiety may contribute to mental health resilience. Accordingly, anxiety should not be regarded solely as a risk factor; rather, it may function as a catalyst for constructive coping strategies under certain conditions. The inverse association between anxiety and suicidal ideation observed in the present study may be interpreted within this broader contextual framework.

Our findings reinforce the notion that both acute and sustained stressors contribute to long‐term mental health consequences in healthcare workers. While exhaustion and high workload are well‐documented contributors to psychological distress in medical professionals, the role of social isolation as a persistent stressor highlights the need for targeted interventions that go beyond workload management. Addressing professional burnout alone may not be sufficient to mitigate the psychological burden experienced by hospital staff. Future efforts should incorporate strategies to foster a sense of connection, peer support, and institutional trust, particularly in prolonged crises such as the COVID‐19 pandemic. Additionally, given the strong association between social isolation and suicidal ideation, mental health support systems should prioritize initiatives that reduce isolation and promote workplace cohesion.

Cultural factors may also have influenced the psychological responses observed in this study. For instance, in Japan, social norms, such as collectivism, emotional restraint, and a strong sense of duty in the workplace, may contribute to prolonged stress responses and a reluctance to express psychological distress. This tendency has been linked to mental health stigma and reduced help‐seeking behaviors among Japanese workers.^49^, ^50^ Moreover, a comparative study found that psychological distress during the COVID‐19 pandemic was lower in Japan than in Western countries, such as the United Kingdom, potentially reflecting cultural differences in emotional expression and perception of stress.^51^ These findings suggest that sociocultural context should be considered when interpreting psychological outcomes and developing support strategies. Future studies comparing multiple cultural settings would help elucidate the role of cultural values in shaping healthcare workers' psychological responses to prolonged crises, such as the COVID‐19 pandemic.

There are some limitations in our study. First, it was a repeated cross‐sectional study, and because the participants were employees working at the same hospitals, replacements occurred to some extent because of transfers and retirements; thus, we were not able to observe longitudinal changes in the same individuals. Second, the participation of these surveys was voluntary and self‐administered, which may introduce selection bias or response bias. For instance, individuals experiencing severe psychological distress may have been less likely to participate, potentially leading to an underestimation of mental health deterioration. However, the same surveys were administered repeatedly to workers at the same two hospitals, and we believe that this study constitutes a valuable report that clarifies the course of the psychological impact of the COVID‐19 pandemic on hospital workers. Third, our study is limited by its sample characteristics, as data were collected from only two hospitals in Japan. While this allows for consistency in the study environment, it also limits the generalizability of our findings to healthcare workers in other regions or hospital settings with different healthcare systems, resource availability, or institutional policies. Differences in workplace culture, mental health support systems, and pandemic response measures may have influenced the psychological impact on healthcare workers in ways not captured by our study. Additionally, both hospitals included in this study admitted patients from the *Diamond Princess* cruise ship during the early phase of the pandemic. This unique and high‐profile exposure may have intensified the psychological responses of staff members at the outset, and should be considered when interpreting the generalizability of our early‐phase findings. Fourth, although Item 9 of the PHQ‐9 is commonly used as a screening tool for suicidal ideation in epidemiological studies, it has limitations in terms of sensitivity and specificity, and may not fully capture the complexity of suicidal thoughts or behaviors.^52^, ^53^ Therefore, the findings related to suicidal ideation in this study should be interpreted with caution. More comprehensive assessment tools may be necessary in future research.

Moreover, although we examined demographic and occupational factors associated with psychological stress and suicidal ideation, several relevant factors were not assessed in detail. Variables such as shift work, workload intensity, and access to mental health resources may have played a significant role in influencing stress levels. Pre‐existing mental health conditions and individual coping strategies, such as resilience and social support, were also not included in our analysis. A more comprehensive assessment incorporating these factors could provide deeper insights into the long‐term psychological impact of the pandemic on different subgroups of healthcare workers and inform targeted mental health interventions. Despite these limitations, our study provides valuable insights into the prolonged mental health impact of COVID‐19 on hospital workers and underscores the importance of sustained institutional and policy‐level efforts to support their psychological well‐being.

In conclusion, this study demonstrated that psychological trauma/stress and suicidal ideation persisted among hospital workers throughout the COVID‐19 pandemic, and that reducing staff fatigue, enhancing knowledge of infections and protective measures, and mitigating feelings of isolation may effectively alleviate psychological stress among hospital workers. These findings suggest that comprehensive supportive measures may be crucial for improving the psychological well‐being of healthcare workers in the post‐COVID‐19‐pandemic era.

## AUTHOR CONTRIBUTIONS

*Study design*: Keiko Ide, Akitoyo Hishimoto, Takeshi Asami. *Data acquisition and analysis*: Keiko Ide, Akira Suda, Asuka Yoshimi, Junichi Fujita, Munetaka Nomoto, Masatoshi Miyauchi, Tomohide Roppongi, Toshinari Odawara. *Manuscript drafting*: Keiko Ide, Akira Suda. All authors approved the final manuscript.

## CONFLICT OF INTEREST STATEMENT

Takeshi Asami is an Editorial Board member of *Psychiatry and Clinical Neurosciences Reports* and a co‐author of this article. To minimize bias, he was excluded from all editorial decision‐making related to the acceptance of this article for publication. The other authors declare no conflicts of interest.

## ETHICS APPROVAL STATEMENT

This study was approved by the Yokohama City University Ethics Committee. The study conforms to the provisions of the Declaration of Helsinki.

## PATIENT CONSENT STATEMENT

N/A.

## CLINICAL TRIAL REGISTRATION

N/A.

## Data Availability

The data used in this study are subject to restrictions due to the presence of potentially identifiable information. However, they can be accessed through the data administrator at Yokohama City University. The data set is stored on the server of the Department of Psychiatry at Yokohama City University and is available to researchers who fulfill the necessary criteria for accessing confidential information.
